# Difficult airway predictors were associated with decreased use of neuromuscular blocking agents in emergency airway management: a retrospective cohort study in Thailand

**DOI:** 10.1186/s12873-021-00434-2

**Published:** 2021-03-25

**Authors:** Jutamas Saoraya, Komsanti Vongkulbhisal, Norawit Kijpaisalratana, Suthaporn Lumlertgul, Khrongwong Musikatavorn, Atthasit Komindr

**Affiliations:** 1 Division of Academic Affairs, Faculty of Medicine, Chulalongkorn University, 1873 Rama IV road, Pathumwan, Bangkok, 10330 Thailand; 2 Department of Emergency Medicine, King Chulalongkorn Memorial Hospital, The Thai Red Cross Society, Bangkok, Thailand; 3 Department of Emergency Medicine, Faculty of Medicine, Chulalongkorn University, Bangkok, Thailand; 4 Department of Internal Medicine, Faculty of Medicine, Chulalongkorn University, Bangkok, Thailand

**Keywords:** Airway management, Rapid sequence induction and intubation, Emergency department, Emergency medicine, Difficult airway

## Abstract

**Background:**

It is recommended that difficult airway predictors be evaluated before emergency airway management. However, little is known about how patients with difficult airway predictors are managed in emergency departments. We aimed to explore the incidence, management and outcomes of patients with difficult airway predictors in an emergency department.

**Methods:**

We conducted a retrospective study using intubation data collected by a prospective registry in an academic emergency department from November 2017 to October 2018. Records with complete assessment of difficult airway predictors were included. Two categories of predictors were analyzed: predicted difficult intubation by direct laryngoscopy and predicted difficult bag-mask ventilation. The former was evaluated based on difficult external appearance, mouth opening and thyromental distance, Mallampati score, obstruction, and limited neck mobility as in the mnemonic “LEMON”. The latter was evaluated based on difficult mask sealing, obstruction or obesity, absence of teeth, advanced age and reduced pulmonary compliance as in the mnemonic “MOANS”. The incidence, management and outcomes of patients with these difficult airway predictors were explored.

**Results:**

During the study period, 220 records met the inclusion criteria. At least 1 difficult airway predictor was present in 183 (83.2%) patients; 57 (25.9%) patients had at least one LEMON feature, and 178 (80.9%) had at least one MOANS feature. Among patients with at least one difficult airway predictor, both sedation and neuromuscular blocking agents were used in 105 (57.4%) encounters, only sedation was used in 65 (35.5%) encounters, and no medication was administered in 13 (7.1%) encounters. First-pass success was accomplished in 136 (74.3%) of the patients. Compared with patients without predictors, patients with positive LEMON criteria were less likely to receive neuromuscular blocking agents (OR 0.46 (95% CI 0.24–0.87), *p* = 0.02) after adjusting for operator experience and device used. There were no significant differences between the two groups regarding glottic view, first-pass success, or complications. The LEMON criteria poorly predicted unsuccessful first pass and glottic view.

**Conclusions:**

In emergency airway management, difficult airway predictors were associated with decreased use of neuromuscular blocking agents but were not associated with glottic view, first-pass success, or complications.

**Supplementary Information:**

The online version contains supplementary material available at 10.1186/s12873-021-00434-2.

## Background

Airway management, especially the use of rapid sequence intubation (RSI), is one of the core competencies of emergency physicians. RSI involves the concurrent administration of induction and paralytic agents to induce relaxation of airway structure to achieve optimal intubating conditions and increase first-pass success [1]. Prior to intubation, the airways must be evaluated for potential difficulty in intubation and oxygenation using bag-mask ventilation. If these possibilities are not properly assessed, the situation of “cannot intubate, cannot oxygenate (CICO)” could arise, which would lead to morbidity or mortality of the patient [2].

Previous studies have identified predictors of difficult intubation in emergency airway management. One of the most researched strategies to predict difficult laryngoscopy and difficult intubation was the use of the mnemonic LEMON [3]. A large observational study identified that the use of modified LEMON criteria provided a sensitivity of 85.7% in predicting difficult intubation by direct laryngoscopy [4]. Patients with high airway assessment scores based on LEMON criteria were associated with a greater chance of a poor glottic view [5].

To minimize the CICO situation, the evaluation of difficult airway risk, including difficult laryngoscopy and difficult mask ventilation, was endorsed by the US National Emergency Airway Management Course [3]. However, it is unclear how patients with these difficult airway predictors are managed in the emergency department (ED) in real world situation. Since difficulty of emergency airway management could result from the devices used [6], intubator experience [7] or the use of neuromuscular blocking agents in RSI [1, 8], it is also unknown how these factors confound the interpretation of the risk of airway difficulty, as they were not commonly explored in previous studies of emergency airway management. It is uncertain how differences in the management of patients with difficult airway predictors lead to different outcomes.

The objective of this study was to explore the incidence, management and outcomes of patients with difficult airway predictors in the ED.

## Methods

### Study design and setting

This is a retrospective study of the data collected by a prospective intubation registry in an ED over a period of one year from November 1, 2017, to October 31, 2018. The study was approved by the ethics committee of the Faculty of Medicine, Chulalongkorn University (IRB No. 068/62). The research was reported according to the Strengthening the Reporting of Observational Studies in Epidemiology (STROBE) Statement.

This study was conducted in an urban, academic ED in a 1500-bed tertiary care hospital. The ED has an annual census of 80,000 visits. There were 8 full-time and 2 part-time board-certified emergency physicians and a total of 27 residents who were enrolled in a 3-year residency training program. The training program in this center was established in 2005. The emergency physicians were responsible for intubation but might have assigned residents or interns to perform intubation under rigorous direct supervision. There were 24-h on-call anesthesia services available in case of suspected difficult or failed airways. The ED was equipped with standard airway equipment, including direct laryngoscope, Macintosh blades of various sizes, laryngeal mask airways, videolaryngoscopes (﻿GlideScope (Verathon, WA, US) and Intubrite (Salter Labs, TX, US)) and a fiberoptic scope (PENTAX FI-13 RBS (PENTAX, Hamburg, Germany)).

### Patients

We included all patients aged 18 or over who presented to the ED and underwent intubation with intubation data recorded in the prospective registry over a 1-year period from November 1, 2017 to October 31, 2018. We excluded patients who were not evaluated for difficult airway risk before intubation.

### Methods and measurements

The data were obtained from a prospective registry of intubation in the ED that was primarily collected for educational purposes by emergency medicine residents. Before intubation, the intubator evaluated the risk of difficult airway and discussed the plan of airway management with the emergency medicine attending. The airway difficulty evaluation result was recorded as either predicted difficult intubation by direct laryngoscopy or predicted difficult bag-mask ventilation. The predictors of difficult intubation are described by the mnemonic “LEMON”, which stands for difficult external appearance (L); the “3–3-2 score”, which includes the mouth opening distance, mandibular space and position of the glottis (E); the Mallampati score (M); obstruction (O); and limited neck mobility (N). The predictors of difficult bag-mask ventilation are described by the mnemonic “MOANS”, which stands for suboptimal mask seal (M); obstruction or obesity (O); advanced age (more than 55 years old) (A); no teeth (N); and stiffness of the lungs (S) [2]. Use of the mnemonics LEMON and MOANS was supported by the national residency training curriculum as a standard way to assess difficult airway in the ED setting. In the present study, patients who were positive for any one component of LEMON or MOANS were categorized as LEMON or MOANS positive. Patients who were either LEMON or MOANS positive were categorized as having a difficult airway. After intubation, the intubator completed the online case record form of the intubation registry. The variables collected were age, sex, weight, diagnosis, reason for intubation, predictor(s) of difficult airway, method of intubation, administration of induction and/or paralytic medicine, number of intubation attempts, operator experience level, glottic view, and complications. The data from the registry were reviewed by experienced residents with the electronic medical records every month to validate data integrity and completeness.

An intubation attempt was defined as attempt of laryngoscopy. First-pass success was defined as successful intubation during the first attempt. Glottic view was defined by the Cormack-Lehane classification system [9]. The sedation method to facilitate intubation was categorized as follows: RSI, which is the administration of both sedative and paralytic agents; sedation without paralysis; or the administration of no medication. The experience level of the operator in the primary intubation attempt was categorized as follows: novice (intern), midlevel operator (first to second year resident) or experienced operator (third year resident or emergency medicine attending).

### Outcome measurement

The primary objective of the research was to determine the incidence of patients with difficult airway predictors in the ED.

The secondary objective was to identify the differences between patients with and without difficult airway predictors in terms of management and outcome. Regarding the management aspect, the use of the backward upward rightward pressure (BURP) maneuver [10], the sedation methods and device used were compared between groups. Regarding the outcome aspect, the glottic view, first-pass success, and complications were compared.

### Data analysis and statistics

Frequency was reported as number and percentage. Continuous data are presented as the mean with standard deviation (SD). The differences in variables were compared using Student’s t-test, the chi-square test or Fisher’s exact test, as appropriate. The binary logistic regression was used for the comparison of the paralytic agent administration adjusted for device used and operator experience. The diagnostic accuracy of LEMON to predict unsuccessful first pass and glottic view grade III or IV was calculated and is presented with sensitivity and specificity. There was no imputation for missing data. However, the number of cases used in the analysis was reported. In our cohort, most patients were of advanced age, which could complicate the interpretation of difficult airway predictors. Sensitivity analysis was conducted to determine whether excluding advanced age as one of the difficult airway predictors in MOANS would affect the interpretation of the analysis. Statistical significance was determined at *p* < 0.05. The analysis was performed with Stata version 16 (College Station, TX, US).

## Results

### Baseline characteristics

During the study period (from November 1, 2017 to October 31, 2018), 226 records were entered in the registry. After the exclusion of 6 records lacking complete evaluation of difficult airway criteria, 220 records met the inclusion criteria. There were 9 studies missing data regarding the glottic view. A total of 220 records were analyzed for the incidence and management of difficult airway, first-pass success and complications. A total of 211 records were analyzed for the difficulty of intubation as categorized by glottic view. The baseline characteristics of the patients in this cohort are shown in Table 1.

**Table 1:** Baseline characteristics of the participants

Characteristics	All patients (*n* = 220)	Without LEMON (*n* = 163)	With LEMON* (*n* = 57)	*p* -value	Without MOANS (*n* = 42)	With MOANS** (*n* = 178)	*p* -value
Age (years)	67 ± 18	66 ± 18	69 ± 20	0.38	43 ± 9	72 ± 15	< 0.001
Male sex	142 (64.5%)	107 (65.6%)	31 (61.4%)	0.56	35 (83.3%)	107 (60.1%)	0.005
BW (kg)	57.8 ± 13	56.9 ± 10.5	60.3 ± 18.2	0.09	58.7 ± 10.3	57.6 ± 13.6	0.6
Diagnosis				0.03			< 0.001
Traumatic cause	6 (2.7%)	4 (2.5%)	2 (3.5%)		3 (7.1%)	3 (1.7%)	
Non-traumatic cause	78 (35.5%)	50 (30.7%)	28 (49.1%)		4 (9.5%)	74 (41.6%)	
Pneumonia							
CHF	41 (18.6%)	35 (21.5%)	6 (10.5%)		3 (7.1%)	38 (21.3%)	
Stroke	27 (12.3%)	24 (14.7%)	3 (5.3%)		12 (28.6%)	15 (8.4%)	
Seizure and alteration of consciousness	19 (8.6%)	16 (9.8%)	3 (5.3%)		6 (14.3%)	13 (7.3%)	
Sepsis	16 (7.3%)	13 (8.0%)	3 (5.3%)		4 (9.5%)	12 (6.7%)	
COPD or Asthma	12 (5.5%)	10 (6.1%)	2 (3.5%)		3 (7.1%)	9 (5.1%)	
Gastrointestinal bleeding	6 (2.7%)	4 (2.5%)	2 (3.5%)		2 (4.8%)	4 (2.2%)	
Upper Airway obstruction	3 (1.4%)	1 (0.6%)	2 (3.5%)		2 (4.8%)	1 (0.6%)	
Other	12 (5.5%)	6 (3.7%)	6 (10.5%)		3 (7.1%)	9 (5.1%)	

Data are presented as mean ± SD or n (percent)

BW: Body weight; CHF: Congestive heart failure, COPD: Chronic obstructive pulmonary disease

*Patients with at least one predictor in LEMON

**Patients with at least one predictor in MOANS

### Incidence of the patients with difficult airway predictors

Difficult airway predictors were present in 183 patients (83.2%); 57 (25.9%) were positive for difficult laryngoscopy predictors (LEMON), and 178 (80.9%) were positive for difficult bag-mask ventilation predictors (MOANS). Difficult external appearance accounted for the largest number of predicted difficult laryngoscopy cases (12.7%), while advanced age accounted for the largest number of predicted difficult mask ventilation cases (72.7%). The details of the difficult airway predictors are presented in Supplementary Table S1.

### Management of the patients with difficult airway predictors

**Fig. 1:**
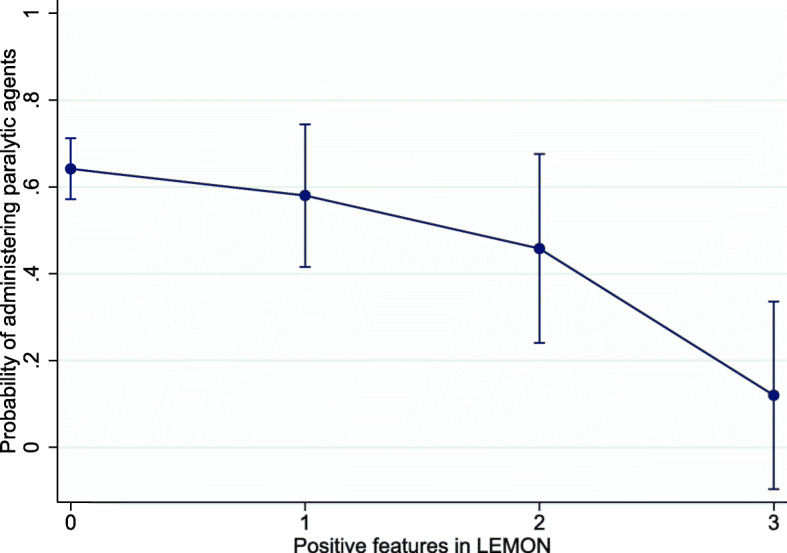
Probability of administering paralytic drugs according to positive LEMON features and adjusted by operator experience and device. The error bars represent 95% confidence interval

There were no significant differences in positioning, BURP maneuver, choice of device or intubator experience between patients with predicted difficult airway and patients without difficult airway predictors (Table 2). Among the patients with at least one difficult airway predictor, RSI was performed in 105 (57.4%) encounters, only sedation without paralysis was performed in 65 (35.5%) encounters, and no medications were administered in 13 (7.1%) encounters. Overall, RSI was used in 131 (59.5%) patients in this cohort. However, in the patients with at least one predictor of LEMON, physicians were less likely to administer paralytic agents [(crude OR (odd ratios) 0.46 (95% confidence interval (CI) 0.25–0.85, *p* = 0.01) and adjusted OR (adjusted for intubating device and operator experience) 0.46 (95% CI 0.24–0.87, *p* = 0.02)] (Table 3). The tendency of administering paralytic drugs decreased as the number of positive LEMON features increased (Fig. 1). No positive association was found with the number of positive MOANS features.

**Table 2:** Management of the patients with difficult airway predictors

Management	All patients (n = 220)	Without LEMON (n = 163)	With LEMON* (n = 57)	*p*- value	Without MOANS (n = 42)	With MOANS** (n = 178)	*p*-value
Sniffing position	171 (77.7%)	127 (77.9%)	44 (77.2%)	0.91	31 (73.8%)	140 (78.7%)	0.5
BURP maneuver	71 (33.2%)	47 (29.9%)	24 (42.1%)	0.1	15 (35.7%)	56 (32.6%)	0.7
Intubation device				0.94			0.82
Direct laryngoscope	186 (84.5%)	138 (84.7%)	48 (84.2%)		35 (83.3%)	151 (84.8%)	
Videolaryngoscope	34 (15.5%)	25 (15.3%)	9 (15.8%)		7 (16.7%)	27 (15.2%)	
1st attempt intubators				0.79			0.8
Novice	72 (32.7%)	55 (33.7%)	17 (29.8%)		15 (35.7%)	57 (32.0%)	
Midlevel	118 (53.6%)	87 (53.4%)	31 (54.4%)		21 (50%)	97 (54.5%)	
Experienced	30 (13.6%)	21 (12.9%)	9 (15.8%)		6 (14.3%)	24 (13.5%)	
Sedation method				< 0.001			0.11
RSI	131 (59.5%)	105 (64.4%)	26 (45.6%)		29 (69.0%)	102 (57.3%)	
Sedation without paralysis	71 (32.3%)	40 (24.5%)	31 (54.4%)		8 (19.0%)	63 (35.4%)	
No Medication	18 (8.2%)	18 (11.0%)	0 (0.0%)		5 (11.9%)	13 (7.3%)	
Pretreatment with fentanyl	29 (13.2%)	19 (11.7%)	10 (17.5%)	0.26	6 (14.3%)	23 (12.9%)	0.81
Induction				0.06			0.27
Etomidate	108 (49.1%)	82 (50.3%)	26 (45.6%)		21 (50.0%)	87 (48.9%)	
Ketamine	67 (30.5%)	46 (28.2%)	21 (36.8%)		10 (23.8%)	57 (32.0%)	
Propofol	19 (8.6%)	13 (8.0%)	6 (10.5%)		6 (14.3%)	13 (7.3%)	
Midazolam	7 (3.2%)	4 (2.5%)	3 (5.3%)		0 (0.0%)	7 (3.9%)	
Paralysis				0.85			0.99
Succinylcholine	122 (55.5%)	98 (60.1%)	24 (42.1%)		27 (64.3%)	95 (53.4%)	
Rocuronium	9 (4.1%)	7 (4.3%)	2 (3.5%)		2 (4.8%)	7 (3.9%)	

BURP: backward, upward, rightward pressure; RSI: rapid sequence intubation

*Patients with at least one predictor in LEMON

**Patients with at least one predictor in MOANS

**Table 3:** Odds ratio of paralytic agent administration in the patients with difficult airway predictors

Difficult airway predictors	Paralytic agents		Odds Ratio (95%CI)	
	Yes	No	Crude	Adjusted*
LEMON (n = 57)	26 (45.6%)	31 (54.4%)	0.46 (0.25–0.85)	0.46 (0.24–0.87)
Without LEMON (n = 163)	105 (64.4%)	58 (35.6%)		
MOANS (n = 178)	102 (57.3%)	76 (42.7%)	0.6 (0.29–1.23)	0.53 (0.24–1.15)
Without MOANS (n = 42)	29 (69.1%)	13 (30.9%)		
MONS (*n* = 77)	37 (48%)	40 (52%)	0.48 (0.27–0.85)	0.37 (0.2–0.7)
Without MONS (*n* = 143)	94 (65.7%)	49 (34.3%)		

*adjusted for device used and operator experience

### Outcomes of the patients with difficult airway predictors

In overall cohort, 43 (19.6%) of the patients had glottic view grade III or IV, and 53 (24.1%) failed first-attempt intubation (Table 4). First-pass success was accomplished in 136 (74.3%) of the patients with at least one difficult airway predictor. There were no significant differences between the groups regarding the glottic view, first-pass success, or complications. No patients underwent surgical airway management. Three patients with difficult airway predictors were complicated by cardiac arrest. Cardiac arrest in these patients did not result from difficult airway, and all three were intubated successfully in the first attempt. In our cohort, the LEMON features was poor predictors of unsuccessful first pass (Table 5).

**Table 4:** Outcomes of the patients with difficult airway predictors

	All patients (n = 220)	Without LEMON (*n* = 163)	With LEMON* (n = 57)	*p*-value	Without MOANS (*n* = 42)	With MOANS** (*n* = 178)	*p*-value
Glottic view				0.94			0.21
Gr I	68 (30.9%)	52 (31.9%)	16 (28.1%)		17 (40.5%)	51 (28.7%)	
Gr II	100 (45.5%)	73 (44.8%)	27 (47.4%)		13 (31.0%)	87 (48.9%)	
Gr III	27 (12.3%)	21 (12.9%)	6 (10.5%)		6 (14.3%)	21 (11.8%)	
Gr IV	16 (7.3%)	12 (7.4%)	4 (7.0%)		4 (9.5%)	12 (6.7%)	
missing	9 (4.1%)	5 (3.1%)	4(7%)		2 (4.8%)	7 (3.9%)	
Attempt of intubation 1 attempt	167 (75.9%)	123 (75.5%)	44 (77.2%)	0.75	32 (76.2%)	135 (75.8%)	0.75
2 attempts	40 (18.2%)	27 (16.6%)	13 (22.8%)		7 (16.7%)	33 (18.5%)	
3 attempts	10 (4.5%)	10 (6.1%)	0		3 (7.14%)	7 (3.9%)	
> 3 attempts	3 (1.4%)	3 (1.8%)	0		0	3 (1.4%)	
Presence of Complications	29 (13.2%)	18 (11.0%)	11 (19.3%)	0.11	4 (9.5%)	25 (14.0%)	0.44
Hypotension	9 (4.1%)	5 (3.1%)	4 (7%)		1 (2.4%)	8 (4.5%)	
Oropharyngeal injury	8 (3.6%)	6 (3.7%)	2 (3.5%)		0	8 (4.5%)	
Hypoxemia	4 (1.8%)	2 (1.2%)	2 (3.5%)		1 (2.4%)	3 (1.7%)	
Cardiac arrest	3 (1.4%)	2 (1.2%)	1 (1.8%)		0	3 (1.7%)	
Mainstem bronchus intubation	2 (0.9%)	1 (0.6%)	1 (1.8%)		1 (2.4%)	1 (0.6%)	
Self-extubation	2 (0.9%)	1 (0.6%)	1 (1.8%)		1 (2.4%)	1 (0.6%)	
Recognized Esophageal intubation	1 (0.5%)	1 (0.6%)	0		0	1 (0.6%)	
Pneumothorax	1 (0.5%)	1 (0.6%)	0		0	1 (0.6%)	
Aspiration	0	0	0		0	0	
Missing	34 (15.5%)	23 (14.1%)	11 (19.3%)		7 (16.7%)	27 (15.2%)	

*Patients with at least one predictor in LEMON

**Patients with at least one predictor in MOANS

**Table 5:** Ability of the LEMON in prediction of unsuccessful first pass or glottic view grade III and IV

	Sensitivity (%) (95% CI)	Specificity (%) (95% CI)	PPV (%) (95% CI)	NPV (%) (95% CI)	AUROC
Unsuccessful first pass	24.5 (13.8–38.3)	73.7 (66.3–80.2)	22.8 (12.7–35.8)	75.5 (68.1–81.9)	0.49 (0.42–0.56)
Glottic view grade III or IV	23.3 (11.8–38.6)	74.4 (67.1–80.8)	18.9 (9.4–32)	79.1 (71.9–85.2)	0.49 (0.42–0.56)

AUROC: area under the receiver operating characteristics; PPV: positive predictive value; NPV: negative predictive value

In the patients with at least one predictor of difficult airway, after adjusting for device and sedation method used, the only factor that increased first-pass success was experienced intubators (*p* = 0.01). Both midlevel intubators (OR 3.2 (95% CI 1.4–7.1), *p* = 0.005) and experienced intubators increased the chance of first-pass success (OR 4.6 (95% 1.2–17.8), *p* = 0.03) compared with novices.

### Sensitivity analysis

In this cohort, 159 out of 220 patients were > 55 years old. A sensitivity analysis of difficult bag-mask ventilation predictors excluding the age factor (i.e., the mnemonic “MONS”) was conducted. A total of 77 patients (35%) were found to be MONS positive. A total of 88 patients (40%) had at least one difficult airway predictor when using LEMON and MONS. We found that there was no significant difference between those with or without MONS predictors regarding glottic view, first-pass success, or complications. However, patients with positive MONS features were less likely to be administered paralytic agents (OR 0.37 (95% CI 0.2–0.7), *p* = 0.002) after adjusting for the device used and operator experience (Table 3).

## Discussion

In this cohort of ED patients who underwent intubation, over 80% were found to have at least one predictor of difficult airway either by direct laryngoscopy or bag-mask ventilation as assessed by the mnemonics LEMON and MOANS, respectively. In the overall cohort, first-pass success was accomplished in 76% of patients, and there were no differences in complications between patients with or without difficult airway predictors. Patients who had predicted difficult intubation by direct laryngoscopy or difficult bag-mask ventilation excluding age factors were less likely to receive paralytic drugs than those who did not. The only factor that was associated with increased first-pass success was intubator experience.

The American Society of Anesthesiologists Task Force of Management of the Difficult Airway reported that there was no standard definition of the difficult airway, but the suggested descriptions included difficult tracheal intubation, difficult laryngoscopy, difficult ventilation, difficult supraglottic airway placement or failed intubation [11]. Previous studies with various definitions of difficult airway identified different incidences of difficult airway in the ED. When using the definition of higher glottis grade, the prevalence of difficult intubation was between 8.1 and 27% [5, 12, 13]. When using the definition of multiple attempts, the prevalence of difficult intubation was between 4 and 29% [4, 7, 14–19]. A meta-analysis of 16 studies found that the first-pass success of all intubations in the ED was approximately 84.1% (95% CI 80.1–87.4%), which means that difficult intubation was approximately 15.9% [20]. Difficult mask ventilation has not been assessed in the ED, but in a very large registry in the operative setting, 0.15% of patients were found to have impossible mask ventilation, and 0.4% had both difficult mask ventilation and difficult laryngoscopic view [21, 22]. In our study, we determined that 19.6% of the patients had difficult airway based on glottic view grade III or IV, and 24.1% had difficult airway based on the definition of multiple attempts. We did not encounter the CICO situation that leads to surgical airway management.

Previous studies that incorporated LEMON reported that the incidence of at least one positive LEMON feature in the ED ranged from 16 to 56% [4, 5, 8, 15]. LEMON was validated as a tool to predict difficult airway in the ED [4, 5, 15]. In contrast to previous studies, our cohort showed that LEMON poorly predicted difficult intubation. The reasons for this disparity might be due to different sedation methods, as fewer patients with positive LEMON features were administered paralytic agents. Moreover, in our study, there were relatively higher proportions of novice operators performing the first intubation attempt than in other studies. Further study should explore the airway difficulty predictors other than LEMON in this specific population.

One situation that emergency physicians would like to avoid is the CICO situation. In traditional RSI, bag-mask ventilation is not recommended due to the risk of gastric distension and aspiration. The assessment of bag ventilation cannot be performed prior to administration of the paralytic agent [3]. Several predictors of difficult bag-mask ventilation have been identified in operating room settings [21, 22]. However, the incidence of CICO was less than 0.5%, and it was unclear whether these predictors could predict CICO in the ED setting. Since, in our cohort, there was no CICO event that led to cricothyroidotomy, the ability of MOANS to predict CICO was not explored. Future studies should include a larger sample size to identify factors that potentially predict difficult bag-mask ventilation in the ED setting.

RSI is endorsed in emergency airway management since it increased first-pass success [1]. Nevertheless, previous studies conducted in different countries have suggested variable rates of RSI usage, especially in difficult airway encounters. In the United States, a multicenter study showed that 68% of all intubations were performed using RSI [16]. In patients with difficult airways, as defined by the presence of multiple predictors of difficult laryngoscopy, the rate of implementing RSI is approximately 80% [23]. In another center in the US, even though 65% of patients had at least 1 predictor of difficult airway, the rate of implementing RSI was 96% [24]. A recent multicenter registry study in the US showed that sedation-assisted intubation was not a common approach when compared with RSI [25]. In Japan, however, the average rate of using RSI was approximately 20% and ranged from 0 to 79% among different centers [19]. In Korea, the rate of RSI use was approximately 56% [8]. In 2009, RSI was used in only 2.75% of intubation cases in the ED in Thailand [17]. In 2012–2016, the rate of implementing RSI increased to 21.6% [12]. In our study, which utilized data collected between 2017 and 2018, the rate was 59.5%. The difference rate of RSI use might be due to the recent adoption of RSI in emergency airway management since emergency medicine is a relatively new specialty [26]. Moreover, our study showed that when patients had positive LEMON features, the tendency to use neuromuscular blocking agents was lower. These findings corroborate those of a previous study in Korea [8]. The avoidance of using neuromuscular blocking agents in patients with anticipated difficult airways was proposed by society guidelines [2, 11]. Regarding this disparity in administering RSI in different settings, further study should focus on the decision making of emergency physicians when to administer neuromuscular blocking drugs.

This study adds information to the paucity of research in emergency airway management in patients with difficult airway predictors. Moreover, we took the confounding factors (e.g., intubator experience and sedation method) into account when the association between first-pass success and difficult airway predictors was analyzed. However, there were several limitations. As we included only intubation managed by emergency medicine services, intubation by other services, though relatively rare, could be missed in this study. In our cohort, we did not collect the detailed explanations of the “L” component in the mnemonic LEMON, such as the presence of beards, secretions, or maxillofacial trauma. Though recommended in the evaluation of difficult airway predictors [2, 3], difficult airway assessment in this study did not include the difficulty of supraglottic device placement or surgical airway access. Nevertheless, in our cohort, no patient needed supraglottic device placement or surgical airway placement. Moreover, the research was conducted in a small number of patients, and thus might not have detected small differences between groups, such as differences in first-pass success or complications.

## Conclusions

In this cohort of airway management in an ED, difficult airway predictors were associated with decreased use of neuromuscular blocking agents but were not associated with glottic view, first-pass success, or complications. Further study should identify physicians’ rationales in deciding whether to administer neuromuscular blocking agents in emergency intubation.

## Supplementary Information

**Additional file 1 Table S1** The presence of difficult airway predictors as categorized by difficult laryngoscopy (LEMON) and difficult bag-mask ventilation (MOANS). The number of patients with each characteristics of difficult airway predictors were showed in Table S1A. The number of difficult airway predictors presented in each patient were presented in Table S1B. **Table S2** Management and outcomes of patients with and without the use of neuromuscular blocking agent during intubation. **Table S3** Glottic view and attempts of intubation in patients with and without positive LEMON features

## Data Availability

The datasets used and/or analyzed during the current study are available from the corresponding author on reasonable request.

## Abbreviations

AUROC: area under the receiver operating characteristics
BURP: backward upward rightward pressure
BW: Body weight
CHF: Congestive heart failure
CI: Confidence interval
CICO: cannot intubate, cannot oxygenate
COPD: Chronic obstructive pulmonary disease
ED: emergency department
IRB: Institutional Review Board
NPV: negative predictive value
OR: odds ratio
PPV: positive predictive value
RSI: rapid sequence intubation
SD: standard deviation
STROBE: Strengthening the Reporting of Observational Studies in Epidemiology
