# Supplementary material for: Difficult airway predictors were associated with decreased use of neuromuscular blocking agents in emergency airway management: a retrospective cohort study in Thailand
Source: BMC Emerg Med. 2021 Mar 25;21:37. doi: 10.1186/s12873-021-00434-2 (PMC7993543; doi:10.1186/s12873-021-00434-2)
Supplement: Supplementary file 1 — Additional file 1 Table S1 The presence of difficult airway predictors as categorized by difficult laryngoscopy (LEMON) and difficult bag-mask ventilation (MOANS). The number of patients with each characteristics of difficult airway predictors were showed in Table S1A. The number of difficult airway predictors presented in each patient were presented in Table S1B. Table S2 Management and outcomes of patients with and without the use of neuromuscular blocking agent during intubation. Table S3 Glottic view and attempts of intubation in patients with and without positive LEMON features [file 12873_2021_434_MOESM1_ESM.docx]

**Supplementary material**

**Table S1** The presence of difficult airway predictors as categorized by difficult laryngoscopy (LEMON) and difficult bag-mask ventilation (MOANS). The number of patients with each characteristics of difficult airway predictors were showed in table S1A. The number of difficult airway predictors presented in each patient were presented in Table S1B.

**Table S1A**

| Difficult airway predictors | n (%) of patients |
| --- | --- |
| LEMON |  |
| L – difficult external appearance | 28 (12.7%) |
| E – evaluation of the mouth opening, mandibular space and position of the glottis | 20 (9.1%) |
| M – Mallampati score | 11 (5%) |
| O – obstruction | 12 (5.5%) |
| N – limited neck mobility | 24 (10.9%) |
|  |  |
| MOANS |  |
| M – suboptimal mask seal | 11 (5%) |
| O – obstruction or obesity | 15 (6.8%) |
| A – advanced age | 159 (72.3%) |
| N – no teeth | 40 (18.2%) |
| S – stiff lungs | 25 (11.4%) |

**Table S1B**

| Number of difficult airway predictors per each patient | n (%) of patients |
| --- | --- |
| LEMON |  |
| 0 | 163 (74.1%) |
| 1 | 30 (13.6%) |
| 2 | 18 (8.2%) |
| 3 | 8 (3.6%) |
| 4 or more | 1 (0.5%) |
|  |  |
| MOANS |  |
| 0 | 42 (19.1%) |
| 1 | 115 (52.3%) |
| 2 | 45 (20.5%) |
| 3 | 15 (6.8%) |
| 4 or more | 3 (1.4%) |

**Table S2** Management and outcomes of patients with and without the use of neuromuscular blocking agent during intubation.

| Characteristics | All patients  (n = 220) | Without  NMBA  (n = 89) | With the  Use of NMBA  (n = 131) | p -value |
| --- | --- | --- | --- | --- |
| Intubation device |  |  |  | 0.046 |
| - Direct laryngoscope | 186 (84.5%) | 70 (78.7%) | 116 (88.5%) |  |
| - Videolaryngoscope | 34 (15.5%) | 19 (21.3%) | 15 (11.5%) |  |
| 1^st^ attempt intubators |  |  |  | <0.001 |
| - Novice | 72 (32.7%) | 15 (16.9%) | 57 (43.5%) |  |
| - Midlevel | 118 (53.6%) | 55 (61.8%) | 63 (48.1%) |  |
| - Experienced | 30 (13.6%) | 19 (21.3%) | 11 (8.4%) |  |
| Glottic view |  |  |  | 0.24 |
| - Gr I | 68 (30.9%) | 26 (29.2%) | 42 (32.1%) |  |
| - Gr II | 100 (45.5%) | 40 (44.9%) | 60 (45.8%) |  |
| - Gr III | 27 (12.3%) | 10 (11.2%) | 17 (13.0%) |  |
| - Gr IV | 16 (7.3%) | 6 (6.7%) | 10 (7.6%) |  |
| - missing | 9 (4.1%) | 7 (7.9%) | 2 (1.5%) |  |
|  |  |  |  |  |
| Attempt of intubation |  |  |  | 0.25 |
| - 1 attempt | 167 (75.9%) | 65 (73%) | 102 (77.9%) |  |
| - 2 attempts | 40 (18.2%) | 16 (18%) | 24 (18.3%) |  |
| - 3 attempts | 10 (4.5%) | 6 (6.7%) | 4 (3%) |  |
| - >3 attempts | 3 (1.4%) | 2 (2.3%) | 1 (0.8%) |  |
| Presence of Complications | 29 (13.2%) | 10 (11.2%) | 19 (14.5%) | 0.39 |

NMBA: Neuromuscular blocking agent

**Table S3** Glottic view and attempts of intubation in patients with and without positive LEMON features.

| Characteristics | All patients  (n = 220) | Without LEMON (n = 163) | 1 positive LEMON feature (n = 30) | 2 positive LEMON features (n = 18) | 3 positive LEMON features (n = 8) | >3 positive LEMON features (n= 1) | P value |
| --- | --- | --- | --- | --- | --- | --- | --- |
| Glottic view |  |  |  |  |  |  | 0.07 |
| - Gr I | 68 (30.9%) | 52(31.9%) | 12 (40%) | 3(16.7%) | 1(12.5%) | 0 |  |
| - Gr II | 100 (45.5%) | 73 (44.8%) | 10 (33.3%) | 11 (61.1%) | 6(75%) | 0 |  |
| - Gr III | 27 (12.3%) | 21 (12.9%) | 3 (10%) | 2 (11.1%) | 1(12.5%) | 0 |  |
| - Gr IV | 16 (7.3%) | 12 (7.36%) | 3 (10%) | 0 | 0 | 1 (100%) |  |
| - missing | 9 (4.1%) | 5 (3.1%) | 2 (6.7%) | 2(11.1%) | 0 | 0 |  |
|  |  |  |  |  |  |  |  |
| Attempt of intubation |  |  |  |  |  |  | 0.95 |
| - 1 attempt | 167 (75.9%) | 123 (75.5%) | 25 (83.3%) | 12 (66.7%) | 6 (75%) | 1(100%) |  |
| - 2 attempts | 40 (18.2%) | 27 (16.6%) | 5 (16.7%) | 6 (33.3%) | 2 (25%) | 0 |  |
| - 3 attempts | 10 (4.5%) | 10 (6.1%) | 0 | 0 | 0 | 0 |  |
| - >3 attempts | 3 (1.4%) | 3 (1.8%) | 0 | 0 | 0 | 0 |  |
